# Temporal Combination Pattern Optimization Based on Feature Selection Method for Motor Imagery BCIs

**DOI:** 10.3389/fnhum.2020.00231

**Published:** 2020-06-30

**Authors:** Jing Jiang, Chunhui Wang, Jinghan Wu, Wei Qin, Minpeng Xu, Erwei Yin

**Affiliations:** ^1^National Key Laboratory of Human Factors Engineering, China Astronaut Research and Training Center, Beijing, China; ^2^Academy of Medical Engineering and Translational Medicine, Tianjin University, Tianjin, China; ^3^Unmanned Systems Research Center, National Innovation Institute of Defense Technology, Academy of Military Sciences China, Beijing, China; ^4^Tianjin Artificial Intelligence Innovation Center (TAIIC), Tianjin, China

**Keywords:** brain–computer interface (BCI), electroencephalogram (EEG), motor imagery (MI), common spatial pattern (CSP), feature selection, support vector machine (SVM)

## Abstract

Common spatial pattern (CSP) method is widely used for spatial filtering and brain pattern extraction from electroencephalogram (EEG) signals in motor imagery (MI)-based brain-computer interfaces (BCIs). The participant-specific time window relative to the visual cue has a significant impact on the effectiveness of the CSP. However, the time window is usually selected experientially or manually. To solve this problem, we propose a novel feature selection approach for MI-based BCIs. Specifically, multiple time segments were obtained by decomposing each EEG sample of the MI task. Furthermore, the features were extracted by CSP from each time segment and were combined to form a new feature vector. Finally, the optimal temporal combination patterns for the new feature vector were selected based on four feature selection algorithms, i.e., mutual information, least absolute shrinkage and selection operator, principal component analysis and stepwise linear discriminant analysis (denoted as MUIN, LASSO, PCA, and SWLDA, respectively), and the classification algorithm was employed to evaluate the average classification accuracy. With three BCI competition datasets, the results of the four proposed algorithms were compared with traditional CSP algorithm in classification accuracy. Experimental results show that compared with traditional algorithm, the proposed methods significantly improve performance. Specifically, the LASSO achieved the highest accuracy (88.58%) among the proposed methods. Importantly, the average classification accuracies using the proposed approaches significantly improved 10.14% (MUIN), 11.40% (LASSO), 6.08% (PCA), and 10.25% (SWLDA) compared to that using CSP. These results indicate that the proposed approach is expected to be practical in MI-based BCIs.

## Introduction

Brain Computer Interface (BCI) is a direct communication system between the brain and external devices, which does not rely on human peripheral nerves and muscles (Wolpaw et al., 2002; Nicolas-Alonso and Gomez-Gil, 2012). In the past few decades, BCI technology has achieved great progress and found a wide range of application scenarios in daily life, such as wheelchair control for disabled patients, entertainment and smart home control for healthy users (Yin et al., 2013; Herweg et al., 2016; Xu et al., 2016). Most of the BCI systems are EEG-based due to its relatively low expense, high portability, better time resolution, and minimal risks to users as compared to other modalities (Arvaneh et al., 2011). At present, the most extensively used brain signals for BCI input are event-related potential (ERP) (Zhang et al., 2014; Jin et al., 2015, 2017; Yin et al., 2015, 2016; Xu et al., 2018), steady-state visual evoked potential (SSVEP) (Pan et al., 2013; Wang et al., 2016; Xing et al., 2018; Zhang et al., 2018), and motor-imagery (MI) (Zhang et al., 2015, 2017; Ang and Guan, 2017; Qiu et al., 2017; Meng et al., 2018; Lugo et al., 2019). MI-BCI works without outer stimuli, thus it is more intuitive for users (Yu et al., 2012; Velasco-Alvarez et al., 2013). Some MI-BCI systems depend on the well-known neurophysiological phenomenon of event-related synchronization (ERS) or event-related desynchronization (ERD), which is either enhancement or suppression of the EEG (Meng et al., 2013). Other MI-BCI systems use slow cortical potentials such as movement-related cortical potentials (Hinterberger et al., 2004; Ren et al., 2014).

The common spatial pattern (CSP) algorithm (Ramoser et al., 2000) is a method that can extract spatial feature for distinguishing the two types of MI tasks in EEG-based MI-BCI systems. The primary goal of the CSP is to compute spatial filters in a data driven manner, which maximizes the difference between the variance of two classes (Arvaneh et al., 2011). However, an important factor on which the effectiveness of the CSP algorithm depends is the specific time segment of the EEG used in the preprocessing phase (Blankertz et al., 2006). This parameter setting has great effect on subsequent CSP feature extraction and classification. Traditionally, a fixed single time period is used as a general setting for the majority of state-of-the-art MI-BCIs, such as a time period of 2.5–4.5 s after optic cue onset (He et al., 2012), 4–7 s or 0–3.5 s from the beginning of trial (Qiu et al., 2016). Although using subject-specific settings can improve the efficiency of CSP approach to a certain degree, these parameters are usually chosen experientially or manually (Blankertz et al., 2006) and may lead to bad experimental results due to the conflict from useless EEG signals.

In this study, a novel temporal combination pattern optimization method is proposed for MI-based BCIs. First, all the motor imagery trials for every type were decomposed to multiple time segments and were processed via band-pass filter on each time segment. Second, the features from multiple time segments of each sample were extracted by CSP spatial filter and were combined to form a new feature vector. Third, the optimal temporal combination patterns for the new feature vector were selected based on the four feature selection algorithms. Last, the classification algorithm support vector machine (SVM) was employed for identifying different categories, then the average classification accuracy was computed via the cross-validation approach. To our knowledge, no research on the incorporation of the feature selection method into temporal combination pattern optimization for motor imagery BCIs has been reported so far.

The remainder of this paper is organized as follows: section Methods describes the proposed methods, including multi-time segmenting and temporal band-pass filtering, EEG feature extraction based on CSP algorithm, feature selection, classification of optimal spatial-temporal features and event-related spectral perturbation; section Results shows description of the datasets and experimental results; section Discussion presents the discussion; and section Conclusion concludes the study.

## Methods

**Figure 1:**
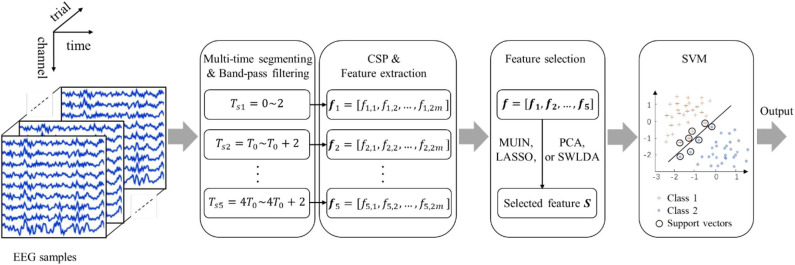
Illustration of temporal combination pattern optimization method.

The proposed temporal combination pattern optimization method is shown in Figure 1. The method mainly includes four parts, which are used for EEG raw data preprocessing, feature extraction and classification (i.e., multi-time segmenting and temporal band-pass filtering, EEG feature extraction based on CSP algorithm, feature selection based on four methods, and classification of selected CSP features). In the training phase, the CSP spatial filters and discriminative CSP features for each time segment are computed using the training data labeled with the two-class MI action. Then the temporal combination patterns are selected, and the SVM classifier model is trained. In the evaluation phase, the class of each single-trial MI task is computed using the parameters obtained from the training phase.

### Multi-Time Segmenting and Temporal Band-Pass Filtering

In the first stage, EEG raw signals are decomposed to a total of five time segments. The time segments are: *T*_*si*_ = *T*_0_(*i* − 1) ~ 2 + *T*_0_(*i* − 1), *i* = 1, 2, ..., 5, where the unit is second, *T*_0_ = 0.5 for dataset 1, *T*_0_ = 0.25 for dataset 2 and 3. When prompted via the optic cue, participants start performing motor imaging tasks. The indexes of these five time windows are represented by the numbers 1 to 5. A third-order Butterworth filter is applied to filter single-trial EEG data for each time segment with frequency range between 8 and 30 Hz. The reason we use these configurations of time segments and band-pass frequency range is that they contain most of empirically chosen parameters in the relevant literatures (Feng et al., 2018).

### EEG Feature Extraction Based on CSP Algorithm

As a spatial filtering approach, CSP has been extensively applied in MI-BCI systems. Its main goal is to minimize the variance of another type of data while maximizing the variance of one type of data, thereby obtaining a set of filters that can extract spatial features (Wang et al., 1999). The variance difference of two-class signals after band-pass filtering is maximized by using CSP projection matrix ***W***. The eigenvectors corresponding to *m* minimum and maximum eigenvalues are used to form a new final filter $W_{2m}\in {ℜ}^{c\times 2m}$. *m* is fixed to 2 in this paper. The features from a trial of EEG signal ***E***are computed as below:

(1) $$\begin{array}{l} f=\log\left(\mathrm{var}\left(W_{2m}^{T}E\right)\right) \end{array}$$

where *f* ∈ ℜ^1 × 2*m*^. Merging of feature matrices from *N*_*s*_ time segments forms a new feature matrix for the *i*th trial as follows:

(2) $$\begin{array}{l} f_{i}=\left[f_{1,i},f_{2,i},...,f_{N_{s},i}\right] \end{array}$$

where $f_{i}\in {ℜ}^{1\times \left(N_{s}*2m\right)}$. The number of time segments *N*_*s*_= 5 in this paper.

To distinguish from the evaluation data, the feature vectors and true class labels from the training data are denoted as $\bar{f}$ and $\bar{y}$, respectively,

(3) $$\begin{array}{l} \bar{f}={\left[{\bar{f}}_{1},{\bar{f}}_{2},...,{\bar{f}}_{n_{t}}\right]}^{T} \end{array}$$

(4) $$\begin{array}{l} \bar{y}={\left[{\bar{y}}_{1},{\bar{y}}_{2},...,{\bar{y}}_{n_{t}}\right]}^{T} \end{array}$$

where ${\bar{f}}_{\;}\in {ℜ}^{n_{t}\times \left(N_{s}*2m\right)}$, $\bar{y}\in {ℜ}^{n_{t}\times 1}$, ${\bar{y}}_{i}$ and ${\bar{f}}_{i}$ denote true class mark and the feature matrix for the *i*th training trial, respectively, *i* = 1,2,…, *n*_*t*_; *n*_*t*_ denotes the total number of training trials.

### Feature Selection

In the third stage, various feature selection algorithms are employed to choose distinguishing feature matrix $\bar{f}$ extracted from training data. Based on the studies performed on the BCI Competition IV Dataset I, III Dataset IVa and III Dataset IIIa, the four feature selection-based algorithms (i.e., mutual information, least absolute shrinkage and selection operator, principal component analysis and stepwise linear discriminant analysis) produced better performance than other feature selection methods (Meier et al., 2008; Ang et al., 2012), so these algorithms are applied in this paper.

#### The Mutual Information-Based Feature Selection Algorithm

Mutual information is a useful measure of information in information theory. It can be seen as the amount of information about a random variable contained in another random variable. The mutual information-based feature selection algorithm is described as follows:

Step 1: Initialize the features ***F***and the selected features ***S***.
  Initialize *S* = ∅, $F=\bar{f}=\left[v_{1}^{T},v_{2}^{T},...,v_{n_{s}\times 2m}^{T}\right]$ from the training data, where $v_{j}^{T}$ is the *j*th column vector of $\bar{f}$.
Step 2: Calculate the mutual information *I*(*v*_*j*_; ω) of every feature *v*_*j*_ using the type tag ω = {1, 2} (Kwak and Choi, 2002).

(5) $$\begin{array}{l} I\left(v_{j};\omega \right)=H\left(\omega \right)-H\left(\omega |v_{j}\right)\\ =-\overset{2}{\underset{\omega =1}{\sum }}p\left(\omega \right)\log_{2}p\left(\omega \right)-\left(-\overset{2}{\underset{\omega =1}{\sum }}p\left(\omega |v_{j}\right)\log_{2}p\left(\omega |v_{j}\right)\right) \end{array}$$

where *p*(ω|*v*_*j*_)log_2_*p*(ω|*v*_*j*_)=$\overset{n_{t}}{\underset{i=1}{\sum }}p\left(\omega |v_{j,i}\right)\log_{2}p\left(\omega |v_{j,i}\right)$, *v*_*j, i*_denotes the *j*th feature of the *i*th trial from *v*_*j*_;*n*_*t*_ is the number of training trials. The probability *p*(ω|*v*_*j, i*_) is calculated via the Bayes theory as follows:

(6) $$\begin{array}{l} p\left(\omega |v_{j,i}\right)=\frac{p\left(v_{j,i}|\omega \right)p\left(\omega \right)}{\overset{2}{\underset{\omega =1}{\sum }}p\left(v_{j,i}|\omega \right)p\left(\omega \right)} \end{array}$$

where *p*(ω) denotes the prior probability of class ω, *p*(ω|*v*_*j, i*_) is the conditional probability of type ω given *v*_*j, i*_,*p*(*v*_*j, i*_|ω) is the conditional probability of feature *v*_*j, i*_ given type ω. *p*(*v*_*j, i*_|ω) can be estimated as follows:

(7) $$\begin{array}{l} \hat{p}\left(v_{j,i}|\omega \right)=\frac{1}{n_{\omega }}\underset{r\in I_{\omega }}{\sum }\phi \left(v_{j,i}-v_{j,r},h\right) \end{array}$$

where *n*_ω_ represents the number of trials belonging to category ω among all training trials, *I*_ω_ represents the set of trials belonging to category ω among all training trials, *v*_*j, r*_ denotes the feature values from the *r*th trial of *v*_*j*_, ϕ represents a smooth Gaussian kernel function, and its smoothing parameter is *h*. The univariate Gaussian kernel is employed as:

(8) $$\begin{array}{l} \phi \left(y,h\right)=\frac{1}{\sqrt{2\pi }}e^{-\left(y^{2}/\left(2h^{2}\right)\right)} \end{array}$$

(9) $$\begin{array}{l} h={\left(\frac{4}{3n_{\omega }}\right)}^{1/5}\sigma \end{array}$$

where σ denotes the standard deviation of *y* in formula (10).

Step 3: Sort all the mutual information *I*(*v*_*j*_; ω) of features in descending order in step 2. Then the first *k*/2 and the corresponding pair of features are selected.

#### LASSO-Based Feature Selection Algorithm

LASSO was first proposed by Robert Tibshirani in 1996 (Tibshirani, 1996), and its full name is Least absolute shrinkage and selection operator. This method is a kind of compression estimation. It obtains a more refined model by constructing a penalty function, so that it compresses some regression coefficients, that is, the sum of the absolute values of the mandatory coefficients is less than a fixed value; at the same time, some regression coefficients are set to zero. Therefore, the advantage of subset shrinkage is retained, and it is a method for processing biased estimates with complex collinearity data (Nigham and Aggarwal, 2005; Meier et al., 2008). The calculation process of the LASSO-based method is as bellow:

Step 1: Initialize the features ***F***and the selected features ***S***as the step 1 of Section 2.3.1.
Step 2: Calculate the contribution degree (*CD*) of every feature *v*_*j*_ with the type tag.
  Given a random variable of the type tag $y={\left[y_{1},y_{2},...,y_{n_{t}}\right]}^{T},y_{i}=\left\{1,2\right\}$ (Meier et al., 2008; Meinshausen and Yu, 2009), a standard linear regression function can be expressed as:

(10) $$\begin{array}{l} y=F\beta +\varepsilon \end{array}$$

where ***y***is a *n*_*t*_ × 1 vector, $F=\bar{f}=\left[v_{1}^{T},v_{2}^{T},...,v_{n_{s}\times 2m}^{T}\right]$ denotes a *n*_*t*_ × (2*mn*_*s*_) matrix, ε denotes a noise vector whose mean is 0 and variance is constant. With reference to (Wang et al., 1999), the estimated value of LASSO can be expressed as:

(11) $$\begin{array}{l} \hat{\beta }=\underset{\beta }{\arg\min}\left(||y-F\beta |{|}_{2}^{2}+\lambda ||\beta |{|}_{1}\right) \end{array}$$

where ||·||_1_, ||·||_2_ represent the *l*_1_-norm and *l*_2_-norm respectively. λ denotes a compensation factor which can encourage a sparse solution LASSO estimate $\hat{\beta }$ (i.e., many entries in $\hat{\beta }$ are equal to zero). By using quadratic programming (Schittkowski, 1985), the solution $\hat{\beta }$of the optimization problem depicted by Eq. (13) can be computed. The entries β_*j*_ in the LASSO estimator $\hat{\beta }={\left[\beta_{1},\beta_{2},...,\beta_{2mn_{s}}\right]}^{T}$ between the class label ***y***and the feature ***F***imply the contribution degree (*CD*) of the *j*th feature *v*_*j*_. Since $\hat{\beta }$ is sparse to some degree, meaning that most of the values in $\hat{\beta }$ are 0, we can classify the *CD*s of different features in the feature set ***F*** to the class label ***y***. The variable *CD*s of diverse features is defined as: *CD*_*j*_ = |β_*j*_|.

Step 3: Sort the *CD*s of features which meet *CD*_*j*_ > 0 in descending order in step 2. Then we choose the first *k* features. If the number of selected features is less than *k*, the penalty parameter λ will be adjusted.

#### Principal Component Analysis-Based (PCA) Feature Selection Algorithm

PCA is a statistical approach that is very effective in linear dimensionality reduction and feature selection. A set of variables that may be correlated is transformed into a set of linearly uncorrelated variables by orthogonal transformation. The transformed set of variables is called the principal component. The PCA-based algorithm is described as follows:

Step 1: Initialization as the step 1 of Section 2.3.1.
Step 2: Calculate the linear transformation ***W***of the feature ***F***given the type tag$y={\left[y_{1},y_{2},...,y_{n_{t}}\right]}^{T},y_{i}=\left\{1,2\right\}$.
  The PCA can maximize the retention variance through a linear transformation*y* = *WF*. In other words, it finds the ***W***by minimizing the reestablishment error. Each row vector of ***W***points to the normalized orthogonal eigenvector computed from the signal covariance matrix. We can use singular value decomposition (SVD) as one simple approach to PCA.
Step 3: For dimensionality reduction, the column vectors of *U*_*F*_corresponding to the *k* largest eigenvalues are chosen to form a final required transformation matrix ***W***.

#### Stepwise Linear Discriminant Analysis-Based (SWLDA) Feature Selection Algorithm

The SWLDA is a commonly used algorithm in pattern recognition. Its main idea is to minimize the distance within the class and maximize the distance between the classes, to obtain the optimal projection direction to produce the best classification results. SWLDA mainly includes two parts, stepwise forward and backward analysis, and weighting the input variables using least squares regression to achieve classification of the type tag ω = {1, 2}. The main flow of SWLDA-based method is as bellow:

Step 1: Initialize the features ***F***and the selected features ***S***as the step 1 of Section 2.3.1.
  Create an initial function that can select features. This function calculates the significance (i.e., *p*-value) via the *F*-test to get the most significant feature that can predict the type tag. The condition that the feature is selected to enter ***S***is set to *p* < 0.1, and the condition to remove from ***S***is set to *p* > 0.15 (Krusienski et al., 2006).
Step 2: A new feature *v*_*j*_is interpolated into the process. By performing the forward stepwise analysis, the largest statistically significant feature will be added into ***F***if *p* < 0.1. By performing the backward stepwise analysis, the least statistically significant feature will be removed from ***S***if *p* > 0.15.
Step 3: Repeat step 2 until the number of selected features in ***S***reaches a predefined number or until the entry/removal condition is not satisfied for all features in the model. We set the maximum number of significant features to *k*.

### Classification of Optimal Spatial-Temporal Features

The fourth part introduces the model building and pattern recognition of the selected feature vectors. It is worth noting that because the selected CSP spatial filters appear in pairs, when using the proposed algorithm for feature selection, if a feature is chosen, its corresponding feature is also chosen. After performing feature selection in the section Feature selection, the selected feature vectors for training EEG signals are denoted as $F\in {ℜ}^{N_{t}\times k}$ where *n*_*t*_ denotes the total number of training trials and *k* is set to 4. *k* = 4 means two pairs of feature vectors are selected, that is, the selected features appear in pairs. We set *k*=4 in this work because the number of features is also 4 (i.e., 2m = 4) for the algorithm without feature selection, which is compared with the four feature selection-based algorithms in the Section Experimental Results.

The support vector machine (SVM) is used in this work since it has broad applications in classification. Plenty of BCI researches reported outstanding performance using SVM for classification. SVM finds a normal vector and a side-play amount of a discrimination hyperplane to separate the data from two classes by maximizing margins between two classes (Meng et al., 2013). LIBSVM is used as the classification tool in the current study (Chang and Lin, 2011).

### Event-Related Spectral Perturbation

The event-related spectral perturbation (ERSP) is a common method that can be used to examine the spectral power changing law of the electroencephalogram from the view of time-frequency domain, which could show ERD/ERS phenomenon of diverse MI tasks. Variable ERSP with frequency and time is defined as follows:

(12) $$\begin{array}{l} ERSP\left(f,t\right)=\frac{1}{N}\overset{N}{\underset{i=1}{\sum }}\left(G_{i}{\left(f,t\right)}^{2}\right) \end{array}$$

where *N* denotes the total number of trials, and *G*_*i*_(*f, t*) denotes the spectral power for *i*th trial at a specific time (*t*) and frequency (*f* ) (Delorme and Makeig, 2004). For each motor imagery trial, we calculate the average spectrum power from −3 to 5 s and between 1 and 35 Hz, where *t* = 0 denotes the time of cue onset. In this article, a two-dimensional plot of ERSP over time and frequency for the three main channels (i.e., C3, CZ, C4) is displayed and used for discussion.

In addition, topographical distribution was displayed to show the distribution of ERD/ERS in different regions of the brain during the execution of different motor imaging tasks. The averaged ERSP value was calculated within alpha band and imagination period (4 s). Since 60 EEG channels (except HEO, VEO, CB1 and CB2) were recorded in the dataset 3, the time-frequency and topographical figures were plotted using the EEG data from participant “l1,” which achieved a relatively high classification accuracy.

## Results

### Description of the Datasets

**Figure 2:**
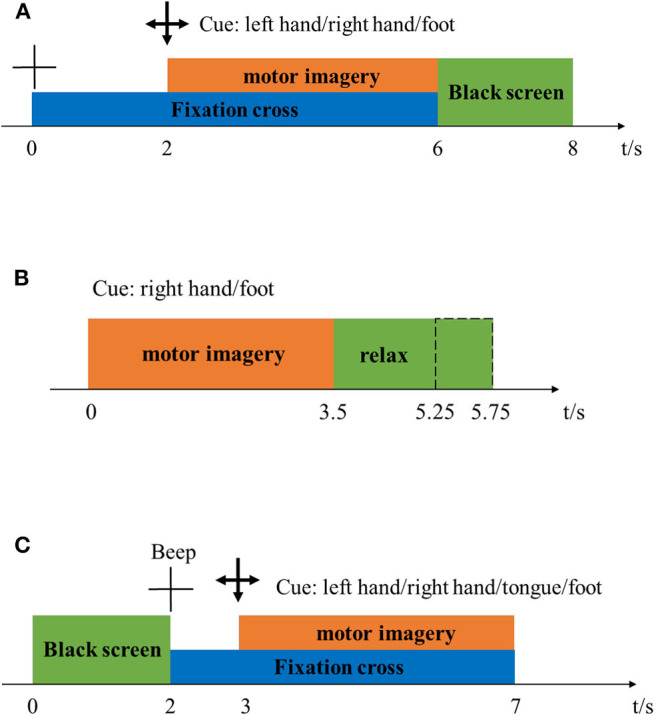
Timeline of one trial in the dataset 1 (subgraph **A**), 2 (subgraph **B**), and 3 (subgraph **C**).

Dataset 1 (BCI Competition IV Dataset I) (Ang et al., 2012): The dataset comprised calibration and evaluation data from seven participants, including four healthy individuals (named “a,” “b,” “f,” “g”) and three artificially generated “participants” (named “c,” “d,” “e”). We only used the calibration data from each participant consisting of two runs, totaling 200 trials for two types of MI tasks where these two types of tasks come from left hand, right hand or foot. Figure 2A shows the timeline of every trial. First, a fixed cross for 2 s appears on the display to prompt the participants to prepare. Then, a 4 s arrow would appear on the display to prompt the participant to start the MI task. The directions of the arrows are left, right, or down, respectively, indicating left, right and foot imagination tasks. Last, the screen was all black for 2 s, indicating that the trial was over. In this process, a fixed cross appeared for a total of 6 s, as shown in the Figure 2A. EEG signals for 59 channels were recorded at 1,000 Hz sampling rate. These data were then bandpass filtered (0.05–200 Hz) and downsampled to 100 Hz. The details of the competition including ethical approval, and the data download website is as follows: http://www.bbci.de/competition/iv/.

Dataset 2 (BCI Competition III Dataset IVa) (Blankertz et al., 2006): The dataset included EEG signal from five healthy participants. Each participant was instructed to complete 280 trials. In each trial, the participants were asked to perform 3.5 s right-hand and foot motor imagery missions. Then participant was instructed to relax for a period of changing length (see Figure 2B). EEG signals for 118 channels were recorded at 1,000 Hz sampling rate. These data were then bandpass filtered (0.05–200 Hz) and downsampled to 100 Hz. The details of the competition including ethical approval, and the data download website is as follows: http://www.bbci.de/competition/iii/.

Dataset 3 (BCI Competition III Dataset IIIa) (Blankertz et al., 2006): The dataset included EEG signals from three subjects labeled “k3,” “k6,” and “l1,” who were instructed to complete 90, 60 and 60 trials, respectively. In each trial, the screen was first completely black for 2 s, during which subjects could rest and relax. Then a “beep” sound was issued, and a cross “+” appeared on the display to remind the participants to prepare for this trial. This process lasted for 1 s. From *t* = 3, a 4 s-long arrow appeared on the screen, prompting the subject to start performing motor imaging tasks. The directions of the arrows were left, right, up, or down, which represented the left, right, tongue, and foot motor imaging tasks (see Figure 2C). EEG signals for 60 channels were recorded at 250 Hz sampling rate. For the purpose of evaluating the feature selection methods of optimal temporal combinations, the EEG signals of left-hand and right-hand motor imagery missions were selected in the current study. The details of the competition including ethical approval, and the data download website is as follows: http://www.bbci.de/competition/iii/.

### Experimental Results

For the above competition data in section Description of the datasets, we made a comparison from multiple angles on the performance among the proposed feature selection of optimal temporal combination patterns algorithms and the traditional CSP algorithm (denoted CSP). The fixed time windows (2–6 s for the dataset 1 and 3–6 s for the dataset 2 and 3 as shown in Figure 2) were employed for analyses. For the proposed algorithms, we used four feature selection-based methods: mutual information, least absolute shrinkage and selection operator, principal component analysis and stepwise linear discriminant analysis (denoted as MUIN, LASSO, PCA and SWLDA, respectively).

The experiment was performed by applying 10 × 10-fold cross-validation to avoid overfitting on the datasets 1–3. In each fold of this procedure, we optimized the temporal combination pattern and trained CSP spatial filters and classification model on the 90% training samples. The remain 10% samples were used as testing data to evaluate the performance of different algorithms. After finishing this procedure for all 10-folds, all data had been involved in the test set.

Table 1 shows the accuracy and significance comparisons of different methods (i.e., CSP, MUIN, LASSO, PCA and SWLDA algorithms) applied on dataset 1, 2, and 3. Experimental results show that classification accuracy is significantly enhanced by using the proposed algorithms compared with the traditional CSP method. Specifically, for three datasets, the proposed feature selection-based methods achieve higher classification accuracies. For seven subjects of dataset 1, the average accuracies are 74.6% (CSP), 90.3% (MUIN), 90.0% (LASSO), 82.9% (PCA), and 88.6% (SWLDA), respectively. For five subjects of dataset 2, the average accuracies are 86.9% (CSP), 87.0% (MUIN), 89.3% (LASSO), 86.9% (PCA), and 89.6% (SWLDA), respectively. For three subjects of dataset 3, the average accuracies are 78.8% (CSP), 82.2% (MUIN), 84.1% (LASSO), 83.5% (PCA), and 82.2% (SWLDA), respectively. The results from three datasets confirm the superiority of the proposed algorithms over the CSP algorithm (paired *t*-tests, *p* < 0.05). Among the four feature selection-based methods, the results show that the LASSO yield best average classification accuracy (88.6%) across all the participants compared to MUIN, PCA, and SWLDA (87.6, 84.3, and 87.7%). By applying a paired sample *t*-test on the datasets 1–3, we find that this superiority is significant (*p* = 0. 03, 0.04, and 0.046). But in these three methods (MUIN, PCA, and SWLDA), the classification accuracy between each pair is not significant (*p* > 0.05). The results from the three competition datasets confirm that the proposed algorithms are significantly better than the CSP algorithm in terms of classification accuracy. Using a paired *t*-test, the resulting *p* < 0.05.

**Table 1:** Accuracy (%) and significance comparisons of different methods applied on dataset 1, 2, and 3.

**Participant**	**Methods**				
	**CSP**	**MUIN**	**LASSO**	**PCA**	**SWLDA**
a	55.5	87.5	86.5	78.0	84.5
b	66.0	82.5	83.0	78.0	82.0
c	77.5	92.0	92.0	66.0	87.0
d	90.5	96.5	98.0	93.5	97.5
e	92.5	100.0	100.0	98.0	98.5
f	85.5	91.5	91.0	90.5	91.5
g	54.5	82.0	79.5	76.0	79.0
Mean ± std	74.6 ± 14.8	90.3 ± 6.3	90.0 ± 7.0	82.9 ± 10.6	88.6 ± 7.0
aa	80.7	81.8	83.6	80.0	83.2
al	97.5	95.7	98.9	98.9	98.9
av	68.2	68.6	70.4	66.1	72.5
aw	95.7	96.8	97.1	96.4	96.8
ay	92.1	92.1	96.4	93.2	96.8
Mean ± std	86.9 ± 11.0	87.0 ± 10.6	89.3 ± 10.9	86.9 ± 12.3	89.6 ± 10.2
k3	85.6	93.3	93.9	92.2	91.7
k6	60.8	57.5	61.7	62.5	60.8
l1	90.0	95.8	96.7	95.8	94.2
Mean ± std	78.8 ± 12.8	82.2 ± 17.5	84.1 ± 15.9	83.5 ± 14.9	82.2 ± 15.2
*p*-value	–	0.0097	0.0016	0.048	0.002

**Figure 3:**
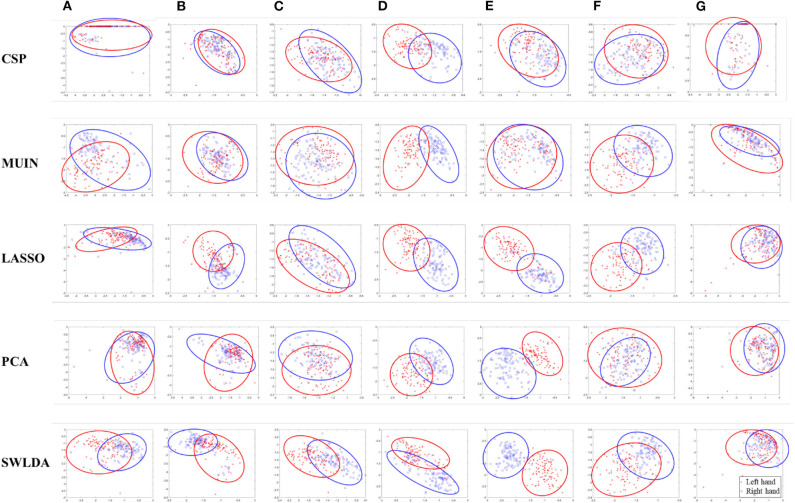
A two-dimensional feature distribution map for each class obtained by using traditional method and the proposed feature selection-based algorithms (i.e., MUIN, LASSO, PCA, SWLDA) in dataset 1 [subjects **(A–G)**].

To explain the superiority of the proposed algorithms, we compared the two-dimensional feature distribution between the methods made with and without feature selection. As shown in Figure 3, we depicted the two-feature distribution of each class in dataset 1 for seven participants “a”-“g.” The red asterisks and blue circles represented the left and right feature types, respectively. For every subject, the sub-graph in the first row indicates that it was drawn directly using CSP, and the sub-graph in the bottom indicates that it was obtained using the proposed algorithms (i.e., MUIN, LASSO, PCA, SWLDA see section Methods). The results from Figure 3 demonstrate that the features selected by the proposed algorithms are easier to distinguish and perform the pattern recognition, compared with the features obtained by traditional method.

**Figure 4:**
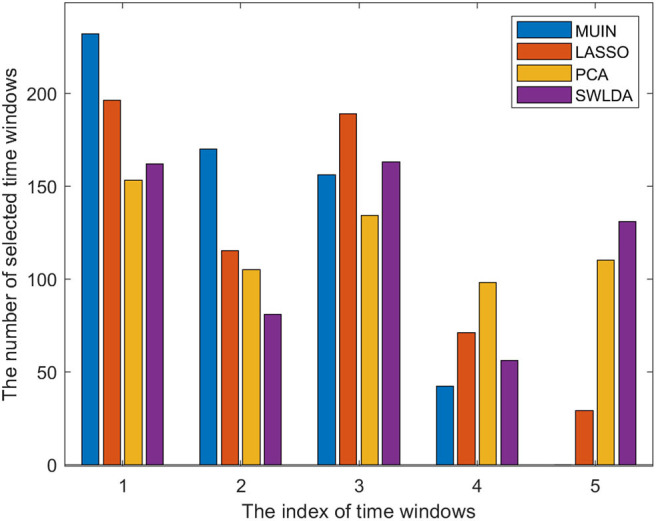
The bar chart represents the total number of selected time windows for the proposed algorithms in datasets 1–3. (The meaning of time window indexes can be found in Section Multi-time segmenting and temporal band-pass filtering).

We also calculated the total numbers of selected time windows based on the proposed methods for datasets 1–3 to determine whether the selected time windows varied in different methods (see Figure 4). The results indicate that the numbers of selected time windows for the proposed methods are different. Interestingly, the number of features selected by the four methods in the time window indexes 1–3 is more than that in the time window indexes 4–5, which indicates that the early period of the motor imagery task contributes more to the classification accuracy.

We further calculated the proportion of samples with selected features from different time windows to the total samples in datasets 1–3 via 10-fold cross-validation. The results of applying different approaches on the data of each participant are shown in the Table 2. The mean ratios are all more than 50%, which shows that the proportion of features selected from the combination of different time windows exceeds the proportion from the same time window for the four methods. More specifically, the ratio for method LASSO is the highest, which explains to some extent that the method LASSO achieves a relatively high average classification accuracy (88.6%). Note that both ratios of participant d in MUIN and participant f in LASSO are zero. It may be because the features from the same time window are sufficient for both cases to provide all the information needed for classification.

**Table 2:** Ratio comparison of samples with selected features from different time windows to the total samples.

**Participant**	**Methods**			
	**MUIN**	**LASSO**	**PCA**	**SWLDA**
a	0.7	0.6	0.6	0.8
b	0.2	0.5	0.9	0.3
c	0.9	1	0.5	1
d	0	0.7	0.5	0.9
e	0.3	1	0.8	0.8
f	1	0	0.8	1
g	0.7	0.7	0.9	0.8
aa	0.7	1	0.9	1
al	1	0.8	1	0.5
av	0.9	1	0.7	0.5
aw	0.6	0.7	0.7	0.4
ay	1	1	0.7	1
k3	1	1	1	0.8
k6	0.7	1	0.5	0.7
l1	0.5	1	0.9	0.7
Mean ± std	0.68 ± 0.3	0.8 ± 0.27	0.76 ± 0. 17	0.75 ± 0.22

## Discussion

As we know, feature extraction plays an important role in motor imagery (MI)-based BCI studies (Park et al., 2013; Kevric and Subasi, 2017). As an effective spatial feature extraction algorithm, the CSP algorithm has been widely applied in MI-BCI related research fields and has achieved admirable results. Recently, some researches have improved and optimized the traditional CSP algorithm to solve the issue of the parameter setting in the time segment of the EEG used. These solutions could be divided into the following two categories.

(1) Automatic selection for parameters. Time segment selection method based on correlation (Feng et al., 2018) is proposed to choose a subject-specific time window for CSP correlation analysis. Efficient wrapper-based methodology (German et al., 2013) is proposed for automatic selection of features computed in different time segments.

(2) Feature selection using information measure. For example, Fisher's common spatial pattern (FCSP) (Fattahi et al., 2013) uses the Fisher's criterion as an optimal function for estimating the spatial and spectral filters. Mutual information-based method (Ang et al., 2012) automatically optimizes the time windows and frequency ranges, by calculating the MUIN variable between the spatial and temporal features reflected by the EEG data and the activity of the corresponding micro-neurons. CSP-tangent space mapping (TSM) algorithm (Kumar et al., 2017) is proposed by utilizing Riemannian tangent space information for extracting features.

Although all the solutions improve the performance of the traditional CSP algorithm to varying degrees, none of them consider the temporal combination patterns during the MI task. From Table 2 we can find that the feature vectors are selected from diverse time windows for most participants. Although the time during which the subject performs the task is known, the time when the brain activity is associated with the task is unknown and it may even be intermittent. Although we know the time when the subjects perform the motor imagery task, the time when ERD / ERS phenomenon occurs is unknown. Therefore, using a fixed single time period for data interception and pattern recognition does not obtain the best classification performance. Although some studies have begun to focus on the optimization of time segment (Ince et al., 2009; Higashi and Tanaka, 2013; Qiu et al., 2016; Feng et al., 2018), the combination of time windows is not considered.

In this study, the proposed feature selection-based method considers the temporal combination patterns among participants' data during motor imagery mission, and applies the feature selection algorithms to optimize the combination patterns of time windows for every participant. We then verified the effectiveness of the proposed methods using competition datasets and obtained significantly higher classification accuracy, compared with the CSP method (see Table 1).

As shown in Figure 1, the band-pass filtering plays an important role in the proposed algorithm and has a significant impact on the final result. Because of the heavy load of computation, it is difficult to employ multiple frequency bands for filtering the EEG measurements in optimization of the frequency-temporal feature. Therefore, a fixed range of band-pass frequencies is used to filter the EEG data. Theoretically, the interaction effect between the time window and the frequency range will affect the classification effect. Therefore, without optimizing the frequency range, optimizing only the time window may not get the best classification results (Xu et al., 2014). Thus, a fixed band-pass frequency range may not be the optimal band-pass filter setting to some extent. In subsequent research work, to solve this problem, we will try to incorporate the multiple frequency bands in the temporal optimization to find optimal combination of frequency band and time segment. Maybe, different subjects have different optimal time segments. In the future, we will try to use other multi-time segments and optimize the multi-time segments for each subject to further improve the performance of the proposed method.

**Figure 5:**
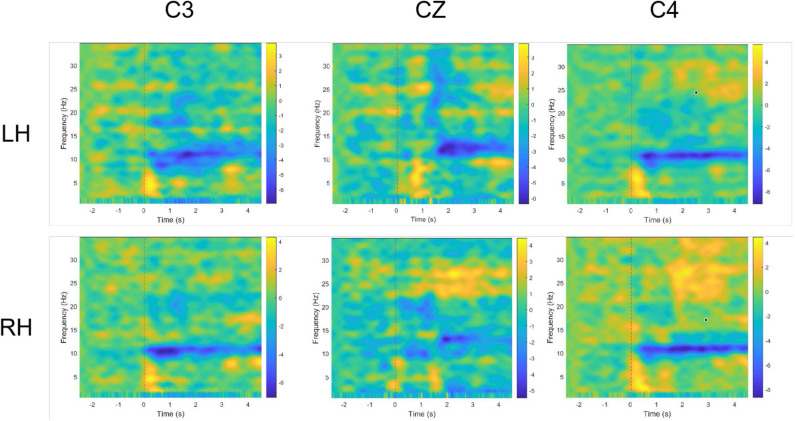
Time-frequency plots for participant “l1” under 2 MI mission and 3 channels (C3, CZ, and C4). LH and RH indicate left hand, right hand, respectively. Blue indicates ERD.

The time-frequency plots of different motor imaging tasks are shown in Figure 5, where blue areas indicate the ERD phenomenon. We used data from the C3, CZ, and C3 channels of the subject “l1”. Left-hand and right-hand MI tasks are represented by LH and RH, respectively. As shown in Figure 5, after the subjects perform the MI tasks for 500 ms, a clear and persistent ERD phenomenon appeared in the alpha band. Under two kinds of motor imagery tasks, the ERD phenomenon appears in the 8–9 Hz frequency band at the C4 channel, and the phenomenon between two tasks is not quite different. In contrast, the ERD phenomenon under the two tasks at the C3 channel is significantly different. Compared with right hand MI task, the ERD feature band of left-hand MI task is broader in alpha rhythm (9–11 Hz). In addition, the ERD in alpha rhythm is intermittent and instable (i.e., sometimes ERD is strong but sometimes is weak). This explains the rationality of temporal combination pattern optimization to some extent.

**Figure 6:**
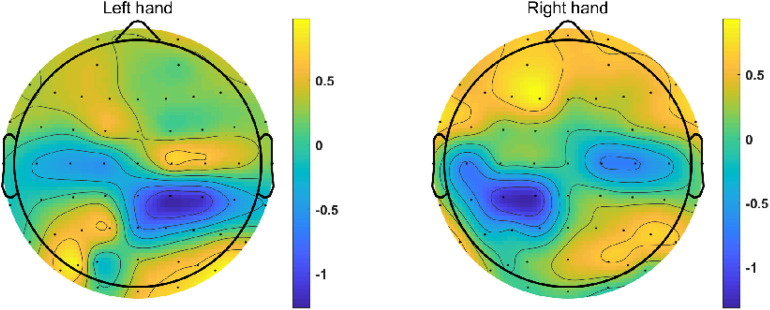
Topographic maps of 2 MI missions from participant “l1”. These graphs are obtained using the ERSP value of every channel and interpolation between the channels. The blue area indicates that an ERD phenomenon occurs in the corresponding brain area when the subject performs the motor imagery task.

Figure 6 presents the topographical distributions of two mental tasks obtained from participant “l1”. We can find a clear ERD phenomenon on near all C3 and C4 channels during both MI tasks, which means the ERD is mainly distributed in the sensorimotor region of the cerebral cortex corresponding to human limbs. Additionally, the ERD of left-hand MI task is stronger in right hemisphere compared to that in left hemisphere, and vice versa.

## Conclusion

In this study, a novel feature selection-based method of optimal temporal combination patterns is proposed for MI-BCI systems. In our method, multiple time segments were obtained from each MI sample. After that, the features were extracted by CSP from each time segment and were combined to form a new feature vector. Finally, the four feature selection algorithms and the classification were applied to evaluate the effectiveness of the proposed method. The results from three competition datasets suggested that the proposed algorithms (i.e., MUIN, LASSO, PCA and SWLDA) could improve the performance compared to traditional feature extraction approach (i.e., CSP). Experimental results showed that the LASSO achieved the highest accuracy (88.58%) among the proposed methods. More specifically, the average classification accuracies using the proposed approaches significantly improved 10.14% (MUIN), 11.40% (LASSO), 6.08% (PCA), and 10.25% (SWLDA) compared to using CSP directly on the datasets 1–3 (*p* < 0.05). The proposed algorithms hold promise for practical applications in MI-based BCIs.

## Data Availability Statement

Information for existing publicly accessible datasets are contained within the article.

## Ethics Statement

Ethical review and approval was not required for the study on human participants in accordance with the local legislation and institutional requirements. Written informed consent for participation was not required for this study in accordance with the national legislation and the institutional requirements.

## Author Contributions

Conceptualization: JJ and CW. Methodology, data curation, and writing—original draft preparation: JJ. Software, formal analysis, and visualization: JW. Validation: JJ, CW, and JW. Investigation: WQ and MX. Resources: EY. Writing—review and editing: JJ, EY, and JW. Supervision: MX. Project administration: JJ and EY. Funding acquisition: EY. All authors contributed to the article and approved the submitted version.

## Conflict of Interest

The authors declare that the research was conducted in the absence of any commercial or financial relationships that could be construed as a potential conflict of interest.
